# Patterns of Animal Rabies Prevalence in Northern South Africa between 1998 and 2022

**DOI:** 10.3390/tropicalmed9010027

**Published:** 2024-01-22

**Authors:** Kgaogelo Mogano, Claude Taurai Sabeta, Toru Suzuki, Kohei Makita, George Johannes Chirima

**Affiliations:** 1 Agricultural Research Council, GeoInformatics Division, Natural Resources and Engineering, 600 Belvedere St., Pretoria 0083, South Africa; 2 Department of Geography, Geoinformatics and Meteorology, University of Pretoria, Pretoria 0028, South Africa; 3 Veterinary Tropical Diseases Department, University of Pretoria, Pretoria 0110, South Africa; 4 World Organisation for Animal Health (WOAH) Rabies Reference Laboratory, Agricultural Research Council (Onderstepoort Veterinary Research), Onderstepoort, Pretoria 0110, South Africa; 5 Department of Environmental and Symbiotic Sciences, Rakuno Gakuen University, Ebetsu 069-8501, Japan; 6 Department of Veterinary Medicine, Rakuno Gakuen University, Ebetsu 069-8501, Japan

**Keywords:** northern South Africa, animal rabies, spatial, temporal, time series

## Abstract

Rabies is endemic in South Africa and rabies cycles are maintained in both domestic and wildlife species. The significant number of canine rabies cases reported by the World Organization for Animal Health Reference Laboratory for Rabies at Onderstepoort suggests the need for increased research and mass dog vaccinations on specific targeted foci in the country. This study aimed to investigate the spatiotemporal distribution of animal rabies cases from 1998 to 2017 in northern South Africa and environmental factors associated with highly enzootic municipalities. A descriptive analysis was used to investigate temporal patterns. The Getis-Ord Gi statistical tool was used to exhibit low and high clusters. Logistic regression was used to examine the association between the predictor variables and highly enzootic municipalities. A total of 9580 specimens were submitted for rabies diagnosis between 1998 and 2022. The highest positive case rates were from companion animals (1733 cases, 59.71%), followed by livestock (635 cases, 21.88%) and wildlife (621 cases, 21.39%). Rabies cases were reported throughout the year, with the majority occurring in the mid-dry season. Hot spots were frequently in the northern and eastern parts of Limpopo and Mpumalanga. Thicket bush and grassland were associated with rabies between 1998 and 2002. However, between 2008 and 2012, cultivated commercial crops and waterbodies were associated with rabies occurrence. In the last period, plantations and woodlands were associated with animal rabies. Of the total number of municipalities, five consistently and repeatedly had the highest rabies prevalence rates. These findings suggest that authorities should prioritize resources for those municipalities for rabies elimination and management.

## 1. Introduction

The aetiologic agent of rabies is a member the *Rabies lyssavirus* (RABV) species of the family *Rhabdoviridae*, order *Mononegavirales*, genus *Lyssavirus* [1], and contains a negative-stranded linear RNA genome. RABV is highly opportunistic and can infect susceptible animals (domestic and wildlife) and humans, which progressively leads to a fatal encephalitis [2,3]. In countries endemic for dog rabies, the number of human fatalities is believed to be under-reported, with the economic losses estimated at $4.7 billion globally [4]. Livestock losses account for a significant portion of the cost of rabies in Africa [5]. Thus, controlling rabies at its source is considered an important public health challenge [6,7], especially in endemic regions of Africa and Asia where free-roaming or ownerless dogs are common. Such a measure will lead to a reduction in livestock and human rabies infections. Therefore, it is crucial to sufficiently strengthen rabies surveillance efforts, establish accurate incidence data, and develop a health strategy to lessen and mitigate the economic burden associated with rabies and its prevalence.

In South Africa, the epidemiology of rabies is confined to two cycles of transmission, namely sylvatic, which involves wildlife host species, and domestic (urban rabies), which involves principally domestic dogs and spillover hosts [8,9,10]. The observed exchange of RABVs between wildlife and domestic host species is worrisome and makes the disease’s epidemiology in the country complex, meaning it is difficult to break transmission cycles [11,12]. Numerous rabies outbreaks associated with the canid RABV variant have been reported in the Limpopo, Mpumalanga, Gauteng, Free State, and North West provinces, making the central to northern areas of South Africa endemic canine rabies regions [8,10,11,13,14]. Notably, Mpumalanga has seen a steady decrease in the number of dog cases since 2010 [15]. The current situation, especially in northern South Africa, suggest a confluence of poor surveillance and inadequate vaccination coverage rates in previous years. The opportunistic nature of the canid RABV variant has resulted in the spread of rabies from hotspots to other areas or its emergence in areas where the disease had not been observed before [16]. A specific example is the dog rabies outbreak that occurred in the Vhembe district of Limpopo Province between 2004 and 2006, possibly initiated by a dog rabies variant genetically linked to jackal rabies viruses from southern Zimbabwe [10]. This observation underscores the transboundary nature of rabies.

Currently, there are no official statistics on dog population numbers in South Africa. However, domestic dogs are the reservoir and primary vector species for the spread of rabies, both in the country and in other African countries [17,18]. The Department of Agriculture, Land Reform, and Rural Development (DALRRD) in South Africa has at several times implemented mass vaccination measures for dogs in the Limpopo, Mpumalanga, and North West provinces, yet numerous rabies cases are still diagnosed in dogs, livestock, and humans. The high dog density rates, particularly in urban and peri-urban townships, and high population turnover rates, with the majority of canines being under 3 years old, are thought to be two of the reasons for the failure of the vaccination initiatives [19]. The low vaccination coverage rate is due to inadequate resources, their inefficient allocation, and under-reporting, making it extremely difficult to establish the actual burden of the disease [9]. On the other hand, the role played by other terrestrial wildlife species such as mongooses, black-backed jackals, and bat-eared foxes as reservoirs for rabies in northern South Africa is not well understood [20,21,22].

To achieve the target of the global tripartite alliance, the World Organization for Animal Health (WOAH), World Health Organization (WHO), and Food and Agriculture Organization (FAO) for Rabies Control’s 2030 target of zero dog-mediated human rabies deaths [23], a clear understanding of the disease patterns and predictions of future outbreaks are required to eliminate dog rabies. In South Africa, rabies is a notifiable disease under the Animal Disease Act of 1984. Currently, the DALRRD is responsible for collecting and reporting epidemiological information on suspected and confirmed animal rabies cases. Human rabies cases must also be reported by the National Institute for Communicable Diseases (https://www.nicd.ac.za/, accessed on 15 September 2023). The local state veterinarian forwards all reports and samples of the rabies suspect to the national rabies reference laboratory, where all cases are later laboratory-confirmed and positive cases are added to the national database.

Independent research groups have undertaken extensive molecular characterization efforts for rabies viruses in South Africa [8,10,11,13,14]. Others have documented the spatial distribution of rabies [24,25,26,27] have undertaken ecological investigations to gain an understanding of rabies within specific locations and at the canine and sylvatic rabies interfaces. However, a time series assessment of rabies outbreaks is missing in South Africa. This is crucial to understand provincial and national rabies trends, including the drivers. In this study, rabies surveillance data obtained from 1998 to 2022 were analyzed to determine spatial and temporal patterns of animal rabies distribution in northern South Africa using all compiled historic and laboratory-confirmed data. Furthermore, we assessed land use and land cover changes for local municipalities with more than 20 confirmed rabies cases throughout the study period. The findings are expected to provide a baseline guide and suggestions for rabies management towards the disease’s control and elimination strategy in the future.

## 2. Materials and Methods

### 2.1. Study Area Background

**Figure 1:**
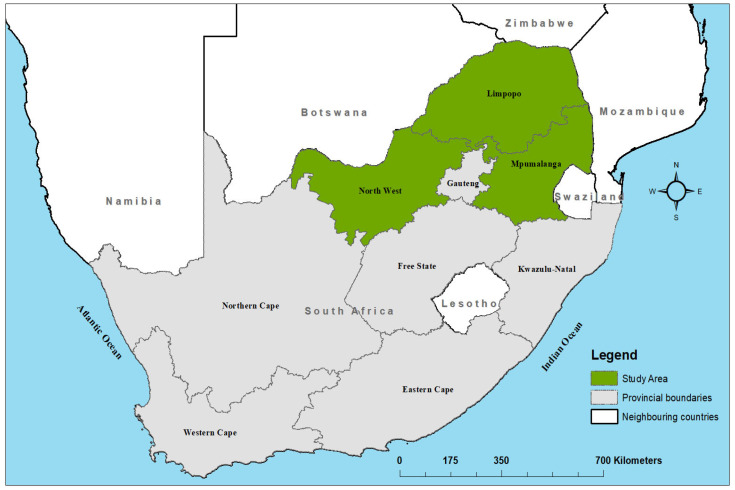
Map of South Africa showing location of the study area.

The study area included three provinces in the northern part of South Africa: Limpopo, Mpumalanga, and North West (Figure 1). Limpopo Province covers an area of approximately 125,754 km^2^, with an estimated human population of 5,405,408 at a density of 43/km^2^. The province has a total of 25 local municipalities. Mpumalanga Province covers an area of 76,495 km^2^, with an estimated human population of 4,039,939 at a density of 53/km^2^. North West Province covers an area of 104,882 km^2^ and has the lowest population of 3,509,953 at a density of 33/km^2^. Both the Mpumalanga and North West provinces have 18 existing local municipalities each (Statistics South Africa Census 2016).

The majority of the human population resides in rural areas where the local economies are primarily agriculturally driven but also rely on tourism and mining [28]. The primary livelihood sources for most people in the study area are seasonal crop farming, livestock farming, government social grants (Perret et al., 2005), and illegal hunting [29]. Domestic dogs are usually kept (1) for security purposes to alert in the case of intruders, (2) to protect and accompany livestock herders, or (3) to protect livestock against predators [29]. Stray dogs are commonly seen near households in the study areas and survive by scavenging or hunting [19]. In the nearby Kruger National Park, cases of stray dogs are infrequently reported at the wildlife and domestic animals interface [27]. In Limpopo Province, blacked-backed jackal species usually come into contact with stray dogs in the villages adjacent to the farming areas [5,10,30,31].

### 2.2. Data Collection

#### 2.2.1. Rabies Data

For this study, domestic and wild animal secondary data sources were collected from the archives of the WOAH Reference Laboratory for Rabies at the Agricultural Research Council—Onderstepoort Veterinary Research (ARC-OVR), Pretoria, South Africa. The database contained 9580 records of specimens submitted for rabies virus infection diagnosis between 1998 and 2022. The epidemiological information recorded for each of the specimens included the date of submission and testing (year, month, and day), diagnosis result (positive or negative), administrative unit (province, local municipality, town or village), geographic coordinates (latitude and longitude in decimal degrees), laboratory reference number, and host species of origin. The samples were then grouped according to their locality of origin (province). The samples that did not have recorded geographic coordinates were assigned the coordinates of the location where the brain specimens originated using a functionality in Google Earth. When the locality of the original specimen was not captured, the state or private veterinary clinic location was used. The geographic coordinates of all submitted specimens were recorded (latitude and longitude in decimal degrees). The results from all the laboratory diagnostic work were imported and converted into shapefiles using ArcMap 10.6 for disease distribution mapping at the local municipality level for visualization.

#### 2.2.2. Diagnoses

The specimens sent to the WOAH Reference Laboratory for Rabies at ARC-OVI were tested using a direct fluorescent antibody (DFA) for the lyssavirus antigen following the guidelines described by [32]. The slides were inspected under ultraviolet fluorescence (Carl Zeiss AG, Göttingen, Germany) after staining with a polyclonal biological conjugate (Bio-Rad, France). The results of the DFA were recorded as positive on a scale ranging from +1 (lyssavirus antigen present in 25% of the fields of the examined smear) to +4 (lyssavirus antigen present in 100% of the fields of the examined smear) or negative (−) if there was no fluorescing antigen.

#### 2.2.3. Land Use/Land Cover Trends and Human Population

In epidemiologic research, sociodemographic and environmental factors have been reviewed to understand the epidemiology of diverse infectious diseases. These factors have yielded valuable insights as predictors of disease occurrence, as demonstrated by [33,34,35,36,37]. Land cover datasets from 2000, 2014, and 2020 were obtained from the Agricultural Research Council—Natural Resource and Engineering (ARC-NRE). Each land cover map was obtained in a raster tiff format. Human population data per local municipality were freely obtained from Statistics South Africa websites (https://www.statssa.gov.za/, accessed on 15 September 2023) for the same years as the land cover data. The three periods were chosen based on the availability of human population data available at the local municipality level. The data between 2003 and 2007 and from 2013 to 2017 were excluded from the spatial analysis, which integrated human population density and land cover data. These periods were excluded due to a lack of data on the human population.

### 2.3. Data Analysis

#### 2.3.1. Descriptive Analysis

We used descriptive statistical tools to characterize and examine potential trends of animal specimens submitted for rabies diagnosis for each of the three provinces. Animal specimens were first categorized into domestic and wildlife species and further sub-categorized into three groups: (1) companion animals (dogs, cats, and horses), (2) livestock animals (cattle, sheep, pigs, and goats), and (3) wildlife species (mongooses, jackals, bat-eared foxes, lions, civets, etc.).

#### 2.3.2. Temporal and Spatial Analysis

Temporal: The data collected over the 25 years were combined into monthly and yearly numbers of incidences, and time series plots were transformed to seasonality and probable trends. The seasonal distribution was determined by summing the frequency rates of rabies incidents by grouping the seasons into rainy (October through March) and dry (April through September). The anticipated numbers of rabies cases were calculated under the hypothesis that the rabies occurrence in northern South Africa is independent of the season and unevenly distributed [38,39,40]. A chi-squared goodness-of-fit test was used to assess the seasonal influence on the occurrence of rabies. The monthly frequency of rabies occurrence was summed by year to examine the annual trends during the study period. To evaluate the correlation among the annual species categories of rabies cases, Pearson’s correlation test was performed. The analysis was implemented in the R software (version 4.2.1) statistical platform using the packages lm, ggplot2, tydverse, lubridate, and dplyr. The primary component of the time series analysis is the ability to identify trends, since these can be used to generate hypotheses regarding the possible causes of such patterns [41].

Spatial: The distribution of rabies in northern South Africa was depicted using a Geographical Information System (GIS; ArcGIS software, version 10.6.1 ESRI, Redlands, CA, USA). Thematic choropleth maps were created to show the 5-yearly distribution of animal rabies in northern South Africa. Any confirmed rabies cases reported from all species were aggregated and then mapped at the local municipality level to provide a general idea of where the animal rabies cases were most concentrated. This approach enables calculations of the temporal and spatial distributions of rabies incidents from different animal species. Furthermore, GIS was used to carry out a global spatial autocorrelation analysis to generate the spatial patterns of cumulative animal rabies at the municipality level over the study period. Global spatial autocorrelation was performed using a global Moran’s index spanning from −1 to 1. Moran’s index = 0 denotes a randomly distributed space, Moran’s index < 0 denotes dispersion within the space, and Moran’s index > 0 denotes implied clustering within the space. To identify the significant clustering of local municipalities from non-significant clustering, the spatial statistics tool that calculates the Getis-Ord Gi* statistical analysis supported by ArcGIS in ArcMap was used [42] was used. The analysis was conducted to display the spatial clustering of local municipalities with higher or lower than expected numbers of confirmed positive cases and positive cases per person per kilometer square. The statistical analysis was performed for the 1998–2002, 2008–2012, and 2018–2022 periods due to a lack of data on human populations for 2003–2007 and 2013–2017, as the data were captured at the community level. For each period, we displayed the population density per kilometer square, total number of categorically positive cases confirmed per local municipality, spatial clustering of each animal category, and spatial clustering with the rate of population density.

#### 2.3.3. Land Use/Land Cover Trends and Human Population

The three land cover raster files obtained were imported into ArcGIS for visualization. Each land cover file was clipped to the size of the study area. All land cover datasets had more than 10 classes represented and were reclassified to remain within thirteen classes for the analysis. The final land cover categories for each time period were indigenous forest, thicket bush, woodland, low shrub land, plantations, cultivated commercial crops, cultivated subsistence crops, settlements, waterbodies, grassland, mines, bare land, and degraded land. The total area occupied by each land cover class within the local municipality was calculated using Spatial Analyst in ArcMap. The local municipalities with more than 20 confirmed positive cases were dichotomized as highly enzootic, less than 20 as low, and those without any positive case as none. This was done in order to understand the environmental factors contributing to high numbers of rabies cases in those municipalities. Based on this, local municipality classified as highly enzootic were coded as “1” and those classified as low or none were coded as “0”. This was used as the outcome variable in the logistic regression analysis. All variables, including the human population density, land cover classes, and coded local municipalities, were subjected to a univariate analysis with logistic regression. The variables with a *p*-value of less than 0.1 were deemed significant and used in the multivariate analysis. In the multivariate analysis, the Akaike information criterion (AIC) was used to select any combination of variables resulting in the lowest AIC.

## 3. Results

### 3.1. Summary Results of Rabies Animal Cases

From 1998 to 2022, specimens from both domestic and wildlife species were diagnosed for rabies at the WOAH Reference Laboratory for Rabies, ARC-OVR, Pretoria. A total of 9580 animal species were confirmed after being laboratory-tested. The overall number of positive rabies cases was 2902 (30.29%), with 6571 negative cases (68.59%), while 107 cases were either unsuitable or gave inconclusive results. Most of the specimen submissions (47.45%; 4546 cases) came from Mpumalanga Province, followed by Limpopo Province with 29.59% (2835 cases). A small fraction of specimens originated from North West Province (22.95%; 2199 cases). Of the total number of positive cases confirmed (2902), Mpumalanga accounted for the majority with 40.42% (1173/2902), followed by Limpopo with 39.83% (1156/2902), while the lowest positivity rate of 19.75% (573/2902) came from North West Province (Table 1).

Among the domestic animals, companion animals were the most commonly infected with rabies virus, totaling 59.71% (1733/2902), while livestock species accounted for 21.88% (635/2902). Among the wildlife species, a high positivity rate was obtained from jackals (287 cases) and followed by mongooses (259 cases), while other wildlife species accounted for 2.17% (63/2902). The total numbers of species submitted and their percentages for all categories are given in Table 2.

### 3.2. Temporal Patterns

**Figure 2:**
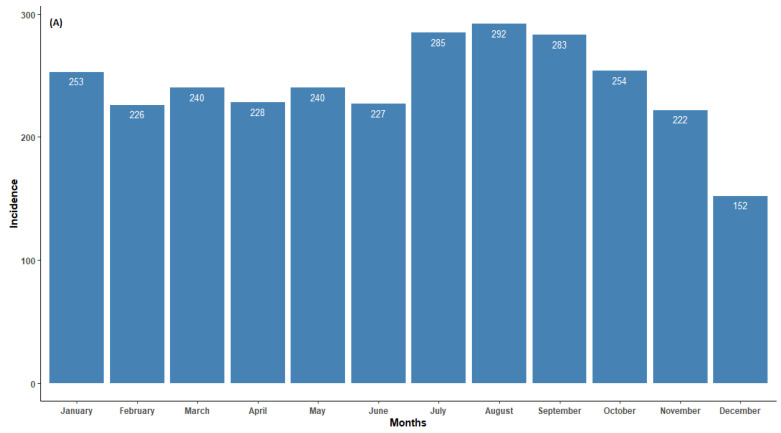

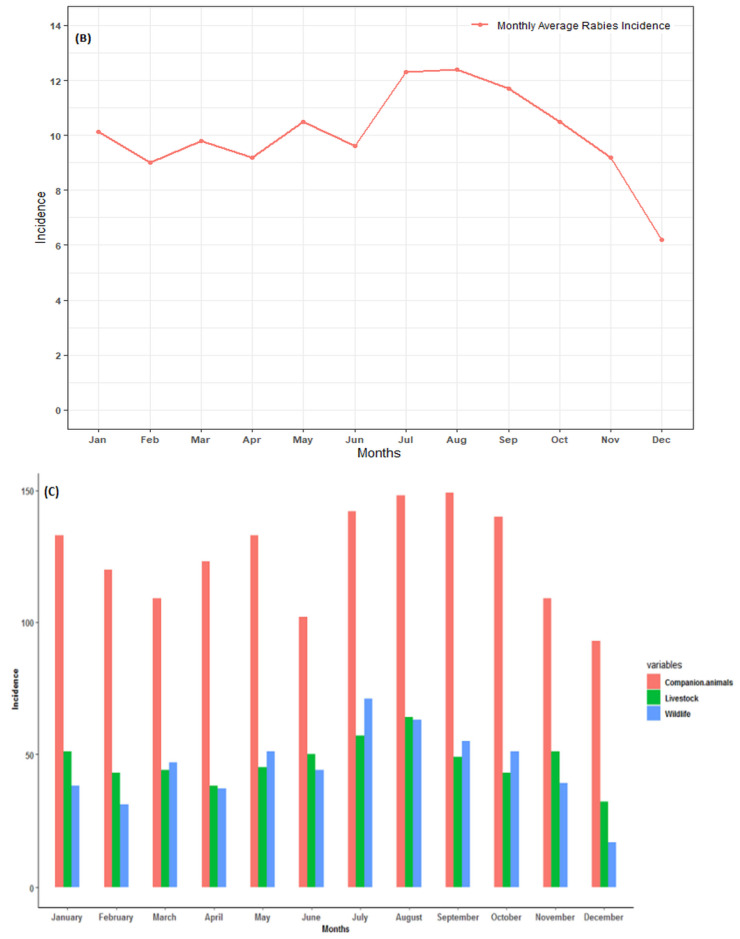
(**A**) Monthly distribution of all laboratory-confirmed rabies cases, (**B**) monthly average of all confirmed rabies cases, and (**C**) confirmed animal rabies cases by categorical species in northern South Africa from 1998 to 2022.

In northern South Africa, animal rabies persisted throughout the year. The incidence of animal rabies peaked in July and August and was relatively low in December (Figure 2). Although increases in cases from companion animals were observed in May, July to October, and January, the seasonal cumulative rabies case numbers largely did not exhibit a clear pattern. The seasonal peak for wildlife was observed in July and started to decrease thereafter. It is worth noting that during the dry season months (specifically June to August), there was an increase in cases from all categories. This is a clear indication that more active surveillance should be performed within these months to understand the social and ecological characteristics of the animal species involved. Seasonality and rabies cases in animals showed a weak negative correlation (r < −0.1). Although there were reduced average occurrence rates of rabies cases in November and December, the seasonality analysis for the original series revealed no statistical significance (*p* > 0.05). This indicates that rabies cases in northern South Africa do not occur seasonally (Figure 2A).

**Figure 3:**
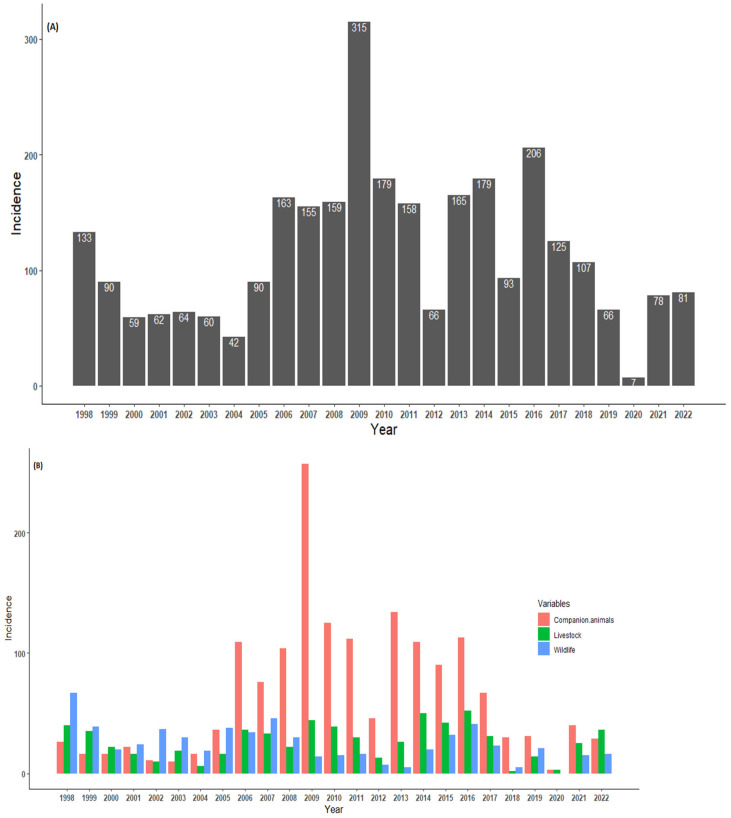
(**A**) Overall annual trends of laboratory-confirmed rabies cases and (**B**) per categorical species in northern South Africa between 1998 and 2022.

The annual trends for laboratory-confirmed animal species are presented in Figure 3. The numbers of confirmed cases in animal specimens were reported throughout the year, with several peaks observed. Between 1998 and 2005, the number of positive rabies cases remained below 200, with a peak observed during 1998 (133 cases, with the majority coming from wildlife species). In 2006 (163 cases), there was a high prevalence rate of confirmed rabies cases. A peak was observed in 2009 (315 cases), with the majority coming from domestic dogs, which was the highest annual peak incidence rate when compared to the focus study. Surprisingly, 2012 showed a decline in the number of positive rabies cases (66 positive cases). Although there was a decline in 2012, the number of cases showed two peaks occurring during the years 2014 and 2016. Thereafter, the number of rabies cases continued to decrease, with the lowest number (7 cases) of the entire study period recorded in 2020. It would be interesting to investigate the sources of the decline and the peaks occurring throughout the study period. Overall, the annual trends were inconsistent in the temporal distribution of confirmed rabies cases. The statistical analysis revealed an increase in the number of rabies cases, although only 20% could be explained by the variations over the years (r2 = 0.24; *p* = 0.04) (Figure 3A).

The annual distribution of categorical rabies cases in animals is presented in Figure 3B. Throughout the study period, it is evident that high numbers of companion animals (domestic dogs) were reported and dominated the trends for the total rabies cases. The rabies cases in all categorical species occurred throughout the year, with several incidence peaks observed. Several obvious peaks occurred in 1998 (wildlife incidence), 2009 (companion animals), 2014, and 2016 for livestock. In 2020, no wildlife species was confirmed as having rabies. It can be observed that between 2006 and 2017, there were more companion species confirmed for rabies. Pearson’s correlation showed a moderate association between companion animals and livestock species (r = 0.5, *p* = 0.005). There was positive weak correlation between livestock and wildlife animals (r = 0.2, *p* = 0.35). However, a negative and non-significant correlation between companion animals and wildlife animals (r = −0.36, *p* = 0.11) was observed.

### 3.3. Spatial Patterns

**Figure 4:**
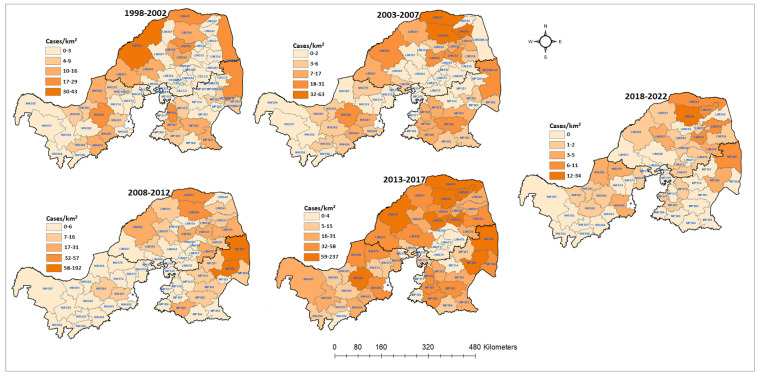
Spatial distribution of cumulative positive animal rabies case numbers in northern South Africa from 1998 to 2022.

The spatial distributions of all confirmed rabies cases in the three northern provinces were mapped by local municipality. From 1998 to 2022, there was a fluctuation in the number of affected municipalities when all diagnosed positive cases were grouped together (Figure 4). Out of the 300 local municipalities, 194 (64%) positive cases of animal rabies were recorded, with the majority of cases confirmed in most parts of Limpopo and Mpumalanga and few in North West. This situation indicates that these areas should be prioritized when planning and allocating resources earmarked for rabies elimination. During the first and last periods of the analysis, there were less than 50 confirmed rabies cases. Between 2008 and 2012, there were only two local municipalities in North West with confirmed rabies cases (15 cases). Furthermore, most of the local municipalities in this province had fewer or no rabies cases confirmed, except in the fourth period of the analysis (2013–2017). There was one municipality (NW384) that consistently had high counts of cases, except in the fifth period of the analysis (2018–2022). However, the opposite was observed in Limpopo and Mpumalanga, with several municipalities constantly reporting rabies throughout the study period. Although there were 5-year period fluctuations in the numbers of animal rabies cases, companion and livestock rabies cases were confirmed more frequently in the Limpopo and Mpumalanga provinces. The majority of the local municipalities with high prevalence rates of confirmed rabies cases are on the border with the neighboring countries of Botswana, Mozambique, and Zimbabwe (Figure 4).

The global autocorrelation in the spatial clustering study revealed that the distribution of confirmed animal rabies cases in northern South Africa was not random, except for the 1998–2002 period (Table 3). The cumulative rabies incident rates during the first and last periods of the analysis varied from 0 to 43 cases per municipality. However, in the first period of the analysis, there was no evidence of geographic clustering based on Moran’s index (0.004, *p* = 0.78), as the cases occurred randomly. Geographic clustering of the cumulative confirmed cases was detected between 2003 and 2022.

**Figure 5:**
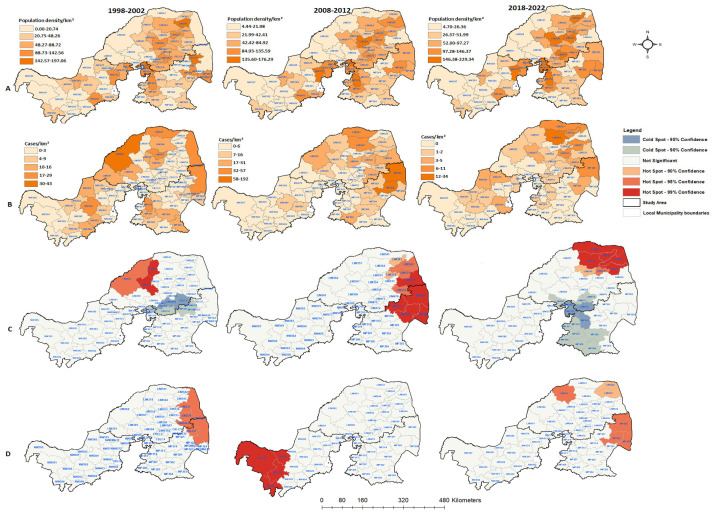
Human population density and spatial distribution of cumulative animal rabies cases over three different time periods in northern South Africa: (**A**) population density rates for three time series per local municipality; (**B**) total number of positive rabies cases per local municipality; (**C**) hot spot and cold spot maps showing spatial clustering of positive cases; (**D**) hot spots and cold spots with the rate of positive cases/population density per local municipality in northern South Africa for 1998–2002, 2008–2012, and 2018–2022.

**Figure 6:**
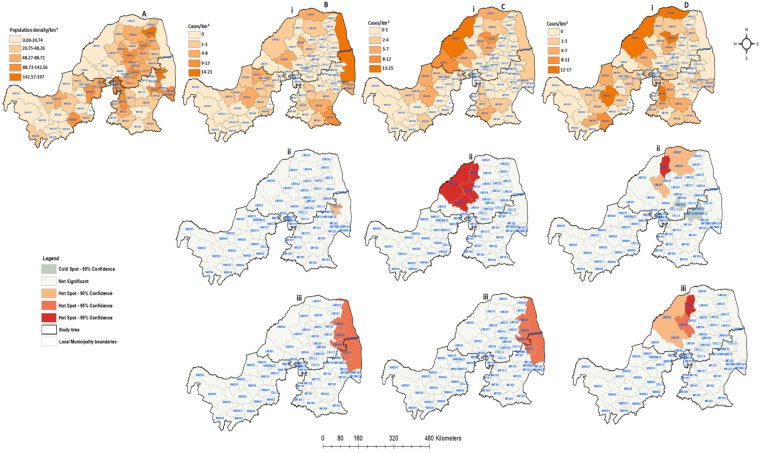
Human population density and spatial distribution of positive confirmed rabies species cases in northern South Africa between 1998 and 2002: (**A**) human population density per square kilometer; (**B**) confirmed companion animals; (**C**) confirmed positive livestock; (**D**) positive wildlife species. All figures with (**i**) represent positive cases per local municipality, (**ii**) hot and cold spots of spatially detected clusters, and (**iii**) hot and cold spots of positive cases with the population density rate.

**Figure 7:**
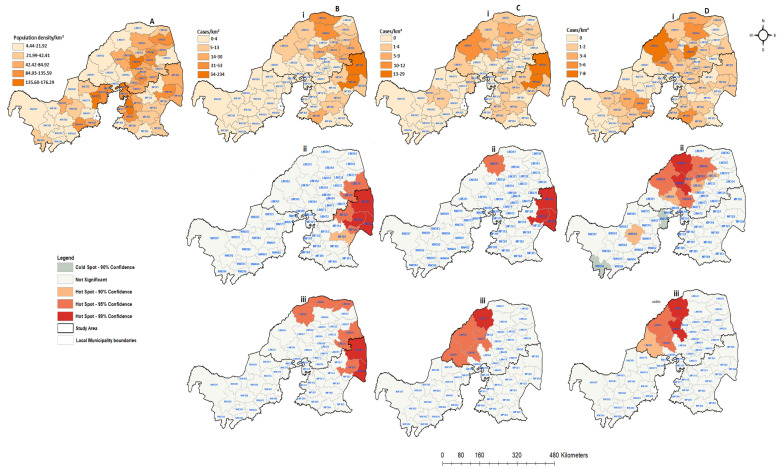
Human population density and spatial distribution of positive confirmed rabies species cases in northern South Africa between 2008 and 2012: (**A**) human population density per square kilometer; (**B**) confirmed companion animals; (**C**) confirmed positive livestock; (**D**) positive wildlife species. All figures with (**i**) represent positive cases per local municipalities, (**ii**) hot and cold spots of spatially detected clusters, and (**iii**) hot and cold spots of positive cases with the population density rate.

**Figure 8:**
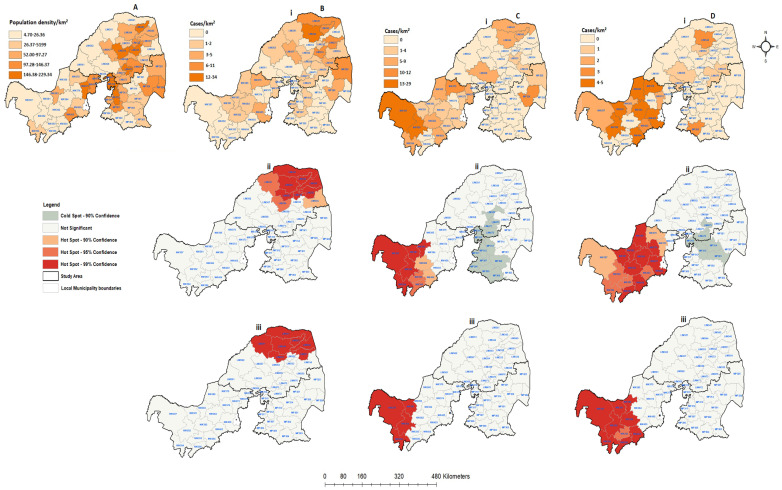
Human population density and spatial distribution of positive confirmed rabies cases in northern South Africa between 2018 and 2022: (**A**) human population density per square kilometer; (**B**) confirmed companion animals; (**C**) confirmed positive livestock; (**D**) positive wildlife species. All figures with (**i**) represent positive cases per local municipalities, (**ii**) hot and cold spots of spatially detected clusters, and (**iii**) hot and cold spots of positive cases with the population density rate.

The results show that the cumulative positive animal rabies rates in northern South Africa are not influenced by increases in human population. The local municipalities with high population densities did not consistently have high confirmed rabies case rates. Furthermore, the spatial analysis also did not detect spatial clusters for the local municipalities with high population densities. During the first time period, hot spots were observed in the western area of Limpopo only (Figure 5C). The cold spots were detected at the boundaries between local municipalities of Limpopo and Mpumalanga. However, during the 2008 and 2012 periods, the hot spot clusters shifted to the east of both provinces, with Mpumalanga having four local municipalities classified as hot spots (*p* < 0.01). When the positive rabies cases were analyzed based on the rate of population density, the results did not follow a uniform pattern (Figure 5D). The significant clustering of positive cases mostly occurred away from densely populated local municipalities. The identified significant hot spots and numbers of positive cases based on the population density per categorical species for each period are depicted in Figure 6, Figure 7 and Figure 8.

The spatial distribution of animal rabies cases, the identified significant hot and cold spots, and the rate of positive cases/population density for each categorical species between 1998 and 2002 were plotted by local municipality (Figure 6). During this period, the majority of local municipalities (22; 37.29%) had a population density of about 20.74 people per square kilometer (Figure 6A). In 59 local municipalities, 23 rabies cases were confirmed in companion animals, 19 in livestock, and 32 in wildlife animals (Figure 6Bi,Ci,Di). The livestock and wildlife rabies cases were shown to have similar spatial patterns of occurrence in Limpopo. A high number of animal rabies cases during this period was reported in local municipalities with low population density rates. The results obtained from the spatial clustering showed that only one municipality with high a number of companion rabies cases was detected (CBLC8) in Mpumalanga. On the other hand, significant spatial clustering (*p* < 0.05) of livestock and wildlife animals could be seen in the eastern areas of Limpopo. There were four local municipalities that showed significant clustering of livestock (*p* = 0.01). When the numbers of positive animal cases were compared to the rate of population density (Figure 6Biii,Ciii,Diii), both the companion and livestock animals showed similar patterns, moving toward local municipalities with high population density rates. The wildlife species also showed clustering dispersing away from the local municipalities with high population density rates. Overall, the three provinces showed confirmed rabies cases from all categorical species.

Figure 7 depicts the spatial distribution of all categorical species and the population density rates between 2008 and 2012 mapped by local municipality, synthesizing the spatial distribution of the passive surveillance efforts over time. The confirmed companion rabies cases were found to be prevalent more in the northern and western regions of the Limpopo and Mpumalanga provinces (Figure 7Bi). During this period, 23 (37.10%) local municipalities did not report any positive rabies cases from companion animals. However, there were two local municipalities from both Limpopo (LIM341 and LIM344) and Mpumalanga (MP325 & MP326) with more than 30 diagnosed positive cases from companion animals. The entire North West Province recorded less than four positive cases. Only 24 (38.71%) local municipalities recorded positive rabies cases from livestock species (Figure 7Ci). The spatial distribution of livestock cases was more prevalent in Limpopo and in the north-eastern part of Mpumalanga, with less than four cases reported in three of the North West local municipalities (NW372, NW383 & NW385). Of the total of 62 local municipalities, only 35 (56.45%) had confirmed wildlife rabies cases, while 27 (43.55%) did not record any wildlife cases during this period (Figure 7Di). During this period, a maximum of 8 positive wildlife cases were recorded. In comparison to companion and livestock animals, North West had increased numbers of local municipalities confirming rabies in wildlife animals, specifically in the central region. The spatial clustering of companion species cases is expected to be significant in the eastern regions of Limpopo and Mpumalanga (*p* < −0.05), corresponding with the actual positive cases. The same pattern was observed for livestock cases in the spatial clustering analysis (Figure 7Cii). The local municipalities adjacent to areas with high positive rabies case rates are also expected to have increased rabies cases rates. Significant wildlife case clusters were detected in the western region of Limpopo, with no spatial clustering observed in Mpumalanga (Figure 7Dii). There were two local municipalities in North West (NW372 and NW394) showing cold spots and one hot spot at the center of the province. Both cold and hot spots in North West Province resulted in a *p* value of 0.1 for wildlife animals. When accounting for the rate of population density (Figure 7Diii), the livestock and wildlife animals were dispersed from the central region of Limpopo, where the population density is high. The same pattern was observed for companion animals. However, in this period, the clusters overlapped, with few local municipalities having high population rates in Mpumalanga. Overall, the population density per square kilometer did not show any influence on the rabies distribution.

Figure 8 displays the population density and spatial distribution of confirmed rabies cases in animals throughout all the three provinces between 2018 and 2022. During this period, the number of confirmed rabies cases was less than 40, the lowest when compared to the previous periods. Of the 57 local municipalities, only 29 (50.88%) showed confirmed companion rabies cases, while 28 (49.12%) did not record any positive companion cases during the entire period (Figure 8Bi). There were 21 (36.84%) local municipalities that recorded livestock rabies cases, with the majority confirmed in North West (24.56%), followed by Limpopo (10.53%) and Mpumalanga (3.51%). Notably, there was a similar distribution of wildlife and livestock rabies cases in North West Province (Figure 8Ci,Di). The results showed that there were sporadic wildlife rabies cases in northern South Africa. However, the disease appeared to be limited in the western region of the study area during this period. Northern Limpopo exhibited high positive companion cases based on the spatial analysis, while no spatial clustering was detected in Mpumalanga or North West (Figure 8Bii). Although the total number of wildlife rabies cases was very low in comparison to other previous periods, North West shows more local municipalities recording positive cases, especially from livestock and wildlife animals (Figure 8Ci,Di). Furthermore, the hot spots areas (*p* < 0.01) were detected in the far west of North West Province, while Limpopo and Mpumalanga remain cold spots for both livestock and wildlife cases (*p* < 0.1). When the positive cases were analyzed with the rate of population density, no significant cluster was observed from Mpumalanga for any categories. The spatial clustering in North West does not overlap with local municipalities with high population density rates. However, in Limpopo Province, only the significant spatial clusters overlap with one local municipality that is high densely populated (Figure 8Biii).

### 3.4. Land Use/Land Cover Trends and Human Population

During the univariate analysis, different predictive land use and land cover variables with *p*-values of less than <0.1 were selected for each period to build a regression model. Waterbodies, grassland areas, thicket bush, and cultivated commercial crops were selected between 1998 and 2002. During the third time period, cultivated commercial crops, cultivated subsistence crops, and waterbodies were chosen for a further analysis. In the last period of the analysis, waterbodies, woodlands, plantations, and degraded land were the variables considered to build a multivariate analysis. Waterbodies was the only environmental variable selected in all three periods. The univariate results did not show any significance of population density for the three periods of the analysis.

In the multivariate analysis, the results indicated that the combination of thicket bush (*p* = 0.030) and grassland (*p* = 0.009) areas gave the lowest AIC (83.74), areas that facilitated high enzootic rabies rates during the first period of the analysis (Table 4). In the third time period, the lowest AIC (59.90) model resulted from the combination of cultivated commercial crops (*p* = 0.023) and waterbodies (*p* = 0.035). The local municipalities with large areas of plantations (*p* = 0.046) and woodlands (*p* = 0.294) had a significantly risk of having a high enzootic rabies rate in the last period of the analysis.

## 4. Discussion

Rabies is a major economic and social concern and is endemic in South Africa, particularly in the northern region, which has been a hotspot since the early 2000s. Rabies cycles are maintained in both domestic and wildlife species, making its control very difficult. The large farming areas and many free roaming dogs contribute to the stability of enzootic rabies [19,31]. Time series and spatial analysis tools can identify and map the prevalence rates of infection. The study’s objective was to evaluate the temporal and spatial patterns of animal rabies cases in northern South Africa from 1998 to 2022, as well as to assess the influence of land use land and cover changes on highly enzootic municipalities.

The analysis showed that dogs accounted for the highest number of rabies cases in the category of domestic animal species. This may be a result of the high number of stray dogs (free-roaming dogs or street dogs roaming the street without having an owner) and semi-owned dogs (dogs with an identifiable owner, which roam the street during the day but have homes to go to and sleep) roaming in the study area [4,43] confirming the maintenance and vector status of this species in southern Africa. The exact dog population remains unknown, and the majority of dog owners do not restrict them from roaming and scavenging the streets. These results corroborate the findings from other areas in Sub-Saharan Africa, where the majority of rabies cases were confirmed in domestic dogs, followed by cattle and then wildlife species (South Africa [25,27,44]; Zimbabwe [45,46]; Namibia [47,48]; Kenya [49]; Zambia [50,51]). Currently, cats constitute less than 2% of companion species confirmed positive for rabies; therefore, they appear not to pose a major public health problem in the region, despite felines being responsible for some human deaths [44,52]. However, cat rabies appears to be an emerging public health problem in North America and South America [53]. It is common for domestic dogs and livestock to have a close relationship, thereby promoting rabies infection, particularly with transmission to cattle (as dead-end hosts). A large number of cattle rabies cases might be attributable to the fact that the Limpopo and North West provinces have both commercial and rural cattle farming practices, and it is in these areas that jackal populations proliferate, thereby maintaining cycles independent of dogs [30] but with an occasional spillover of infection to cattle. In other nations in the region, rabies cases in livestock are commonly linked to wildlife animals [54,55]. Of the wildlife species, only 12 cases of rabies in bat-eared foxes were confirmed between 1998 and 2022. This is because most reviewed reports showed that bat-eared foxes are endemic in Northern Cape Province in South Africa [8,56,57]. Overall, the number of wildlife species submitted for testing outnumbered the samples obtained from livestock species. The role of wildlife in the maintenance and transmission of rabies was explained by [25]. The author found that a high density of jackals was responsible for the majority of rabies cases in wildlife animals, making it a key factor in the maintenance and spread of rabies.

This study suggested that rabies persisted all year round in northern South Africa (Figure 2 and Figure 3), with more than 100 cases reported annually between 2006 and 2017. The reported cases showed a significant upward change between 1998 and 2022. There were surges between 2006 and 2017, except in 2012 and 2020, when the number of cases significantly declined. The apparent stability and reduction in the number of confirmed cases from 2000 to 2004, 2012, and 2020 was positive and may have been connected to the increased control measures at these times. A few possible reasons could have led to the decline in laboratory-confirmed rabies cases in the country, with the primary reason being the lack of funds allocated for diagnostic laboratories. Another reason could be based on management decisions to shift resources from rabies to other disease outbreaks such as malaria, cholera, and COVID-19, especially in 2020, when COVID-19 hit South Africa.

Although annual rabies peaks were observed in companion, livestock, and wildlife animals, the monthly distributions did not show any strongly defined patterns. However, a higher proportion of positive cases in all species was observed from May (mid-dry season) through September (early months of the wet period). A possible explanation is that during the rainy season animals are less likely to socialize as a result of contact among the species, whereas during the dry season their movement follows the spatial patterns of resource availability and creates an opportunity for interaction. Several authors have previously reported seasonality in animal behavior and movement [50,58,59,60]. Although some rabies cases in companion animals seem to be associated with the incidence rates of rabies in livestock and wildlife species, this appears to be only a moderate association, suggesting that there may be a complex relationship that cannot be fully explained by our current data. The temporal patterns of infection in livestock, other wildlife, and companion species depend on chances for contact between these animals and reservoir species [61]. Importantly, the prevalence of rabies in livestock plays an important factor for estimating the economic impact of the disease [62,63]. However, studies on livestock rabies have not been thoroughly undertaken in northern South Africa, and few papers have reported on its economic impact. The high percentage of livestock rabies cases found to be linked to companion species (mainly dogs) corroborates the findings of similar studies undertaken by independent research groups in Bhutan and the Serengeti region of Tanzania. According to those studies, it is possible that dogs are the principal carriers of livestock rabies [40,43,64]. Nonetheless, we can assume that rabies may not always be spread by dogs and that the sylvatic transmission of rabies may occur in any situation involving livestock and wild animal encounters. Other countries have reported rabies infection cases in livestock from wildlife species [65,66]. The animal population density, food availability, infectious diseases, the climate, human activities, and animal management practices influence these elements [62].

The spatial distribution of rabies showed that cases were documented in all three provinces, albeit sporadically. Although rabies outbreaks do not occur simultaneously in every region, in northern South Africa the disease remains endemic. In the spatial analysis, local municipalities that were hot spots for animal rabies in the 25 years of the study period were identified. The majority of the clusters were spread over the western and eastern local municipalities during the study period. The geographical overlap between domestic and wild carnivore species and the corresponding time periods suggest that spillover between the two groups occurs now and again. It is important to take note of the cluster’s proximity to the neighboring countries of Botswana, Zimbabwe, and Mozambique, as this raises the possibility that rabies may have crossed borders and spread from a population of wild animals to a population of domestic animals. Our results are in agreement with the findings from Bhutan, where the districts bordering India were at a higher risk of rabies than the interior district [67]. The findings in Brazil by [68] also demonstrated the risk for the maintenance of the rabies virus in both domestic and wild species that circulate across the country’s borders. It is important that surveillance monitoring is undertaken by all neighboring countries simultaneously to stop the spread of the disease.

The clusters from all categorical species were not constant over time, and it is possible that the cluster sites will change in the future. Previous research has shown that the rabies virus may spread between 30 to 50 km per year [69]. The population density per square kilometer did not increase with the increasing number of confirmed rabies cases. When the positive cases were analyzed with the rate of population density, the results showed that local municipalities with high population density rates did not overlap with the hot spot clusters. Our findings are in contrast to the findings by [48] in Namibia. The authors reported increasing confirmed rabies case numbers in dogs and domestic ruminants with increasing human population density rates. In-depth research is required to comprehend the dynamics of rabies transmission in these regions of northern South Africa at small scales. These findings suggest that rabies management efforts and the allocation of resources toward rabies elimination should be prioritized to the municipalities that consistently reported repeated rabies cases throughout the study period. Furthermore, mass vaccination campaigns should be implemented more often in those areas. The main goal of surveillance is to provide actionable information so that decision-makers can lead and manage more effectively [70,71].

The land use and land cover changes indicated that different environmental variables were positively associated with municipalities constantly reporting rabies cases during different periods of the analysis. Different landscapes retain different vegetation types and waterbodies in different seasons. For instance, during the rainy season, there is plenty of food, water, and shelter for animals. Animals tend to feed where food is readily available and accessible with minimal contact with other host species. In addition, the animals know when to stop feeding and move to other localities [72,73]; and consequently, due to the reduced contact, rabies transmission is unlikely to occur. However, during the dry season, resources are very scarce and competition becomes high among animal species, leading to rapid contact and high rabies transmission rates [74,75]. It was reported that when food and water become scarce during the dry season, the contact rates between black-backed jackal populations rise along with their home range [47,68,76]. Areas characterized by swamps and wetlands and near to river banks retain little water, and certain grasses respond quicker to moisture compared to landscapes characterized by clay and rocks. Therefore, where there is little green grass and water, animals feeding are at high risk of transmission. Changes in land cover, in turn, alter the habitat of reservoir hosts [77,78]. Other studies have found that black-backed jackals are typically found in commercial farms and bushveld areas in the north and central parts of Limpopo Province [10,79]. During the dry season, northern South Africa receives little to no rain. Agricultural intensification, urbanization, and deforestation are the major drivers of land use change, which poses an increased risk in the spread of infectious diseases [77,78,80].

## 5. Conclusions

Several limitations of this study should be taken into consideration. The information was gathered by passive surveillance; therefore, it is possible that there were still many unreported cases of rabies during the study period. Most importantly, the results showed how rabies hotspots could be identified using the current data in a resource-constrained country, making it easier to allocate limited resources for rabies prevention and control in municipalities that predominantly report rabies cases. To meet the “one health” strategy and Sustainable Development Goal 3 by 2030, which aims to “ensure healthy lives and promote well-being for all ages”, urgent appropriate control strategies are required, specifically in the local municipalities identified as being highly enzootic. Precise information from state or private veterinarians’ could aid in understanding the constant clusters in some municipalities and the few instances of rabies in other municipalities. Furthermore, the lack of data on vaccination coverage rates, the number of animals vaccinated annually, the number of animals responding positively towards vaccines, and the period that the vaccination program was implemented made it challenging to understand the decline in the number of rabies cases reported in some other years and the large spatial distribution. Another challenge was the lack of dog population numbers and cattle data, which prohibited us from calculating the ratio of dogs to cattle per municipality. There was evidence of hotspots from all categorical species throughout the study period, despite variations in the numbers of suspected cases across the three provinces.

## Figures and Tables

**Table 1:** Summary results for all specimens submitted and tested for rabies virus between 1998 and 2022 across the three provinces in the study area.

Province	Total Submitted (%)	Negative (%)	Positive (%)	Inconclusive (%)
Limpopo	2835 (29.59)	1660 (25.26)	1156 (39.83)	31 (28.97)
Mpumalanga	4546 (47.45)	3317 (50.48)	1173 (40.42)	56 (52.34)
North West	2199 (22.95)	1594 (24.26)	573 (19.75)	20 (18.69)
Total	9580	6571 (100)	2902 (100)	107 (100)

**Table 2:** The total positive numbers of animal species submitted from 1998 to 2022 in northern South Africa.

	Categories (*n*)	Animal Species (*n*)	Confirmed Cases (%)
Domestic animals (6866)	Companion animals (5005)		1733 (59.71)
		Dogs (4342)	1576 (54.31)
		Cats (603)	48 (1.65)
		Equine (56)	22 (0.76)
	Livestock animals (1861)		635 (21.88)
		Cattle (1647)	537 (18.50)
		Sheep (63)	17 (0.59)
		Pigs (26)	8 (0.28)
		Goats (126)	73 (2.52)
Wildlife animals (2714)	Wildlife animals (2714)		621 (21.39)
		Mongoose (541)	259 (8.92)
		Jackals (1121)	287 (9.89)
		Bat-eared fox (877)	12 (0.41)
		* Other wildlife (178)	63 (2.17)
	Total (9580)		2902 (30.29)

* African civet (*Civettictis civetta*), honey badger (*Mellivora capensis*), baboon (*Papio*), wild dog (*Lycaon pictus*), antelope (*Bovidae*), zebra (*Equus quagga*), lion (*Panthera leo*), and duiker (*Cephalophinae*). All wildlife species included in the other wildlife species categories totaled less than 30 samples for the duration of the study period.

**Table 3:** Global spatial autocorrelation of cumulative confirmed rabies cases in northern South Africa from 1998 to 2022.

Period	Moran’s I	Z-Score	*p*-Value
1998–2002	0.004479	0.267113	0.789
2003–2007	0.213372	2.980477	0.002878
2008–2012	0.127508	3.045964	0.002319
2013–2017	0.232307	3.990029	0.000066
2018–2022	0.204465	4.508940	0.000007

**Table 4:** A multivariate analysis of risk factors associated with rabies occurrence (>20 rabid animals) in northern South Africa.

Period	Variables	*p*-Values	AIC
1998–2002	Thicket bush + Grassland	0.030 *	83.74
		0.009 **	
2008–2012	Cultivated commercial crops + Waterbodies	0.023 *	59.90
		0.035 *	
2018–2022	Plantations + Woodlands	0.046 *	62.13
		0.294	

Significant at * *p* < 0.05, ** *p* < 0.001.

## Data Availability

Data on rabies diagnoses that support the findings of this study are available upon request from the Agricultural Research Council—Onderstepoort Veterinary Research, Pretoria, South Africa (https://www.arc.agric.za/arc-ovi/Pages/ARC-OVI-Homepage.aspx). The data cannot be shared publicly due to ethical restrictions. The shapefiles for South African boundaries can be obtained freely from the Municipal Demarcation Board (https://www.demarcation.org.za/). The human population data were obtained from Statistics South Africa websites (https://www.statssa.gov.za/). The land Cover data were obtained from the Agricultural Research Council—Natural Resource and Engineering Archives (https://www.arc.agric.za/arc-iscw/Pages/ARC-ISCW-Homepage.aspx). Data on rabies diagnosis that support the findings of this study are available upon request from the Agricultural Research Council—Onderstepoort Veterinary Research, Pretoria, South Africa. The data cannot be shared publicly due to ethical restrictions.
